# Supplementary material for: A synthetic control evaluation of the use of pulse oximeters in response to the COVID-19 pandemic in England
Source: PLoS One. 2025 Dec 5;20(12):e0337617. doi: 10.1371/journal.pone.0337617 (PMC12680260; doi:10.1371/journal.pone.0337617)
Supplement: Attachment — Submitted filename: Response to Reviewers 27.05.25.docx [file pone.0337617.s004.docx]

# Response to Reviewers

We would like to acknowledge the Associate Editor and peer-reviewer for taking their time and offer their considerations to improve and strengthen our submission. We have considered all provided comments and included revisions to the original manuscript to address them for further consideration.

We have provided our response below and uploaded the revised manuscript (clean and edited versions) for further publication consideration.

Yours sincerely,

Dr Stefano Conti (on behalf of all authors)

## Associate Editor

1. [Counterfactual Validation]

Your clarification regarding diagnostic tests (placebo tests, latent factor inspection, MSE, and BIC) is noted. While these details strengthen the methodological rigor, their absence in the manuscript limits transparency. A brief mention of these validation steps in the Methods or Discussion section (e.g., “Placebo tests and model diagnostics confirmed the robustness of the GSynth estimates”) would reassure readers without exceeding word limits.

We thank the Associate Editor for this remark. We have included a sentence at the end of the Methods section mentioning statistical validation work we carried out before upholding inferences from the proposed fitted GSynth models. Word count limitations and the fairly standard nature of much of said diagnostics led us not to elaborate on their nature.

2. [Sensitivity to Alternative Methods]

Your rationale for not pursuing alternative methods (e.g., DiD, RD) is understandable given data limitations and prior consortium efforts. However, explicitly acknowledging these considerations in the Limitations section would enhance the manuscript. For example: “While we considered alternative causal inference approaches (e.g., Regression Discontinuity), data sparsity and heterogeneous implementation precluded their application. Future studies with richer data may explore these methods.”

We thank the Associate Editor for this suggestion. We added an item in the bulleted list the Strengths and limitations of this study section consist of, explaining how we felt that limitations in programme data and operationalisation led us away from adopting causal modelling frameworks other than GSynth. Of note, these considerations were already included in the Discussion section of the original manuscript draft.

3. [Data Sparsity and Bias]

Your justification for using GSynth to mitigate bias is valid. The manuscript already highlights low uptake as a limitation, but explicitly linking this to the choice of GSynth (e.g., “GSynth was selected for its ability to address unobserved confounders, critical given the sparse data”) would strengthen the narrative.

We thank the Associate Editor for this comment, which we factored in by augmenting the paragraph in the Methods section describing the GSynth model with the approach’s ability to pick up potential unobserved confounders.

4. [Interpretability of Results]

The focus on ATT over feature-level interpretability is acceptable. However, a sentence in the Discussion could clarify this scope: “Our analysis prioritized estimating average treatment effects rather than disentangling individual covariate interactions, which may be explored in future patient-level studies.”

We thank the Associate Editor for this remark, and have accordingly included a period in the Methods section specifying how the main goal of the causal analysis was in deriving effect estimates, not in quantifying the influence of available covariates on examined outcome trends.

## Peer-reviewer

We understand that the manuscript alterations previously outlined in the Associate Editor section should address concerns and comments also raised by the peer-reviewer.
