# Supplementary material for: A synthetic control evaluation of the use of pulse oximeters in response to the COVID-19 pandemic in England
Source: PLoS One. 2025 Dec 5;20(12):e0337617. doi: 10.1371/journal.pone.0337617 (PMC12680260; doi:10.1371/journal.pone.0337617)
Supplement: Attachment — Submitted filename: Response to Reviewers 06.10.25.docx [file pone.0337617.s005.docx]

# Response to Reviewers

We would like to acknowledge the Associate Editor and peer-reviewer for taking their time and offer their considerations to improve and strengthen our submission. We have considered all provided comments and feedback and included revisions to the original manuscript for them to be addressed by further consideration.

We have provided our response below and uploaded the accordingly revised manuscript (clean and edited versions) for further publication consideration.

Yours sincerely,

Dr Stefano Conti (on behalf of all authors)

## Peer-reviewer

Q1) Clarify onboarding thresholds and their empirical justification.

A1) We thank the peer-reviewer for this observation. In response to it we expanded on the paragraph in the Results section already dealing with this aspect of the analysis design, explaining the guiding rationale for what is unavoidably a subjective, if data-driven, decision and its consequences on securing a reasonable enough size of comparison groups for GSynth model fitting purpose. This is in addition to considerations already expressed at the end of the Results section around the sensitivity of findings from the main analysis to altering such thresholds.

Q2) Address selection bias from exclusion of CCGs with incomplete data.

A2) We thank the peer-reviewer for raising a very valid point. CCGs submitting part or no returns to the AHSN designated for collection were invariably under severe strain from clinical management of the pandemic, as well as under different data sharing agreements with their respective data collection infrastructure providers. We were told in multiple evaluation sit rep meetings that this fractured information governance set-up posed important limitations to the already reduced data sharing capability at several participating CCGs. We reckon that it is unlikely for the above two limiting factors to have systematically affected to a degree relating to programme delivery the ability of onboarding CCGs to communicate full programme data. We have now included these considerations into the corresponding paragraph of the Discussion section touching on this important limitation.

Q3) Incorporate diagnostics for GSynth model fit and placebo tests in the supplement.

A3) Regrettably this is no longer possible: as outlined in the gsynth package on-line documentation (URL: https://cran.r-project.org/web/packages/gsynth/gsynth.pdf, p. 6), GSynth model instances subsuming the requested information also retain a copy of the data-set the respective model is fitted to. Conformant to the UK Government’s COPI Notices expiry, by 22 June 2022 we were therefore required to remove from our servers all these model objects. Unfortunately, like also indicated in the manuscript, the proposed analysis is no longer reproducible; not even to us. We are sorry for what we have consistently recognised as being an important practical limitation to our outlined work that is independent on our volition.

Q4) Add implementation fidelity measures (if available) or conduct subgroup analyses by delivery model.

A4) We thank the peer-reviewer for pointing us to the notion of implementation fidelity: a framework we were unaware of but nevertheless recognise its usefulness. We have now amalgamated in a redrafted Discussion section our considerations around the facets of programme fidelity, whereby we conclude that we have no reasons to believe that it would have downplayed to a significant extent the already weak evidence of impact transpiring from our (sparse) data analysis. Crucially the availability of sparse data, and our analysis sampling unit being the CCG, explain our practical inability to carry out any subgroup analysis; this limitation is now explicitly outlined in the Discussion section.

Q5) Provide a more structured rationale for the observed null effects, possibly using power calculations or comparisons to other concurrent interventions (e.g., COVID Virtual Wards).

A5) We thank the peer-reviewer for suggesting an interesting analysis angle. We find it genuinely hard to elaborate further, or provide additional reasoning, on the null significance of impact estimates derived from our analysis above and beyond the four points – the fourth of which (around programme fidelity) we thank the peer-reviewer for bringing to our attention – we tease out in the Discussion section. As such we have left the relevant text unchanged; we by all means would be happy to incorporate reflections from any additional suggestion the peer-reviewer may have to corroborate our interpretation. Besides, we did not carry out any power calculation exercise as we set out to inform our evaluation with as many complete data as possible; this is typically the reason why sample size determination very rarely equips evaluations based on retrospectively collected, observational data. In retrospect, such an exercise would have been futile given the severely low programme enrolment our evaluation had to deal with. Lastly, we should note that we never had access to data around, or any involvement with, the entirely independent COVID virtual ward programme.
